# Neutron scattering in photosynthesis research: recent advances and perspectives for testing crop plants

**DOI:** 10.1007/s11120-020-00763-6

**Published:** 2020-06-02

**Authors:** Gergely Nagy, Győző Garab

**Affiliations:** 1 Neutron Scattering Division, Oak Ridge National Laboratory, Oak Ridge, TN 37830 USA; 2 Institute for Solid State Physics and Optics, Wigner Research Centre for Physics, POB 49, 1525 Budapest, Hungary; 3 Institute of Plant Biology, Biological Research Centre, POB 521, 6701 Szeged, Hungary; 4 Department of Physics, Faculty of Science, Ostrava University, Chittussiho 10, Ostrava – Slezská, 710 0 Ostrava, Czech Republic

**Keywords:** Macro-organization, Neutron scattering, Regulatory mechanisms, Structural flexibility, Thylakoid membrane

## Abstract

The photosynthetic performance of crop plants under a variety of environmental factors and stress conditions, at the fundamental level, depends largely on the organization and structural flexibility of thylakoid membranes. These highly organized membranes accommodate virtually all protein complexes and additional compounds carrying out the light reactions of photosynthesis. Most regulatory mechanisms fine-tuning the photosynthetic functions affect the organization of thylakoid membranes at different levels of the structural complexity. In order to monitor these reorganizations, non-invasive techniques are of special value. On the mesoscopic scale, small-angle neutron scattering (SANS) has been shown to deliver statistically and spatially averaged information on the periodic organization of the thylakoid membranes in vivo and/or, in isolated thylakoids, under physiologically relevant conditions, without fixation or staining. More importantly, SANS investigations have revealed rapid reversible reorganizations on the timescale of several seconds and minutes. In this paper, we give a short introduction into the basics of SANS technique, advantages and limitations, and briefly overview recent advances and potential applications of this technique in the physiology and biotechnology of crop plants. We also discuss future perspectives of neutron crystallography and different neutron scattering techniques, which are anticipated to become more accessible and of more use in photosynthesis research at new facilities with higher fluxes and innovative instrumentation.

## General introduction

Neutron scattering (NS) techniques are widely used for the structural and dynamical characterization of condensed matter. Neutrons—due to their electric neutrality—can penetrate deeply into most samples. Due to the nature of their interaction with the nuclei, they are especially sensitive to light atoms while exhibiting different scattering cross-sections for different isotopes of the same atom. Of particular importance for biology, the large difference in scattering length between hydrogen and deuterium allows contrast variation experiments, highlighting and hiding different constituents, e.g. lipids and proteins. Considering also their non-invasive nature, neutron scattering techniques are ideally suited for structural (Engelman and Moore 1972; Stuhrmann 1974; Svergun and Koch 2003; Chen et al. 2012) and dynamical (Doster et al. 1989; Zaccai 2000; Zaccai et al. 2016) studies of biological samples in their functional states (Fitter et al. 2006).

The structural details obtained from neutron scattering techniques range from a few Ångströms, accessible with neutron protein crystallography (see e.g. (Lu et al. 2019)), to nanometer size information, obtained in neutron diffractometers (see e.g. (Demé et al. 2014)), and tens of nanometers, using small-angle neutron scattering (SANS) (Sadler and Worcester 1982; Ünnep et al. 2014a). With different accessible lengthscales, different neutron scattering techniques can help to better understand the atomic structure of proteins, uncover the dynamics of complex molecular assemblies and reveal changes in long-range order of extended proteoliposomes and membrane systems. In this mini-review, our attention will be focused on recent advances on the use of neutron scattering techniques in photosynthesis research, mainly, but not exclusively, SANS investigations. Results and potential applications of less frequently used techniques, such as quasielastic neutron scattering (QENS) and neutron spin echo (NSE), will also be briefly discussed. Regarding the technical details and theoretical backgrounds, we refer the readers to a recently published book chapter (Nagy et al. 2014a).

## Small-angle neutron scattering

By measuring elastically scattered neutrons at small angle, SANS has been used for long in structural biology to study structural features of soft matters at a mesoscopic (1–100 nm) scale. It offers information about the shape and size of macromolecules and molecular assemblies in solution, as well as about the morphology of fibrillar and lamellar structures (Neylon 2008), including samples from photosynthetic organisms.

SANS have been successfully employed to provide size and shape information about micelles of hydrated chlorophylls and chlorophyll mixtures (Worcester et al. 1986, 1989) correlation between the spectroscopical and structural features of supramolecular assemblies (Tiede and Thiyagarajan 1996) or small aggregates of bacteriochlorophyll (BChl) *a* and *c* studied in benzene-d_6_ solutions (Wang et al. 1997). SANS was also used to correlate the aggregation state of the photosynthetic reaction centre-cytochrome c_2_ protein complex with the cytochrome oxidation kinetics (Tiede et al. 2000) or to determine plausible models for the minimal functional unit of the purple bacterial antenna complexes LH1 and LH2 (Tiede and Thiyagarajan (1996). SANS studies on the D_2_O solution of the B820 subunit of the LH1 antenna complex (from *Rhodospirillum rubrum*) demonstrated the benefits of contrast matching allowing the separation of the scattering signals from that of the protein complex and the attached predeuterated detergent (Wang et al. 2003). The main light-harvesting complex of PSII (LHCII) was also successfully studied with contrast variation SANS measurements (Cardoso et al. 2009) providing a low-resolution structure for the protein complex stabilized via detergent in solution which was consistent with the X-ray crystallographic structure of trimeric LHCII. In the thermophylic green phototrophic bacterium, *Chloroflexus aurantiacus* SANS provided information about the size and shape of the chlorosome, the light-harvesting B808-866 complex and the reaction center (Tang et al. 2010, 2011; Tang and Blankenship 2012). SANS can also facilitate the development of successful crystallization protocols of photosynthetic membrane proteins (Thiyagarajan and Tiede 1994). (For a more detailed overview on this area of molecular aggregates and protein complexes, see (Nagy et al. 2014a)).

Most recently, SANS allowed determination of the low-resolution solution structure of the active state of orange carotenoid protein (OCP) and revealed its structural similarity to a stable mutant version of the protein, rendering the latter a potential structural analogue for the light-activated OCP (Golub et al. 2019a); this enabled the authors to characterize the molecular dynamics of the ground and active states of OCP on the picosecond timescale (Golub et al. 2019b).

During the past decade, SANS studies focused on the structure and flexibility of multilamellar thylakoid membrane systems under different experimental conditions and in a variety of photosynthetic organisms. The thylakoid membrane system was first studied with neutrons by Worcester (Worcester 1976) and Sadler and Worcester (Sadler and Worcester 1982), who observed the diffraction peak signal arising from the periodic organization of isolated thylakoid membranes with repeat distances (RDs) consistent with electron microscopy (EM) data. The technical developments in the past decades brought considerably higher neutron fluxes and better resolution of the neutron beams, and higher sensitivity and resolution of the detectors. These, together with the availability of user-friendly sample environments, with magnets, thermostats and sample illumination, and with a large number of mutant organisms impaired in their photosynthetic function as well as the easy-to use tools to characterize the physiological state of the samples, opened new possibilities for the use of SANS in photosynthesis research.

**Fig. 1:**
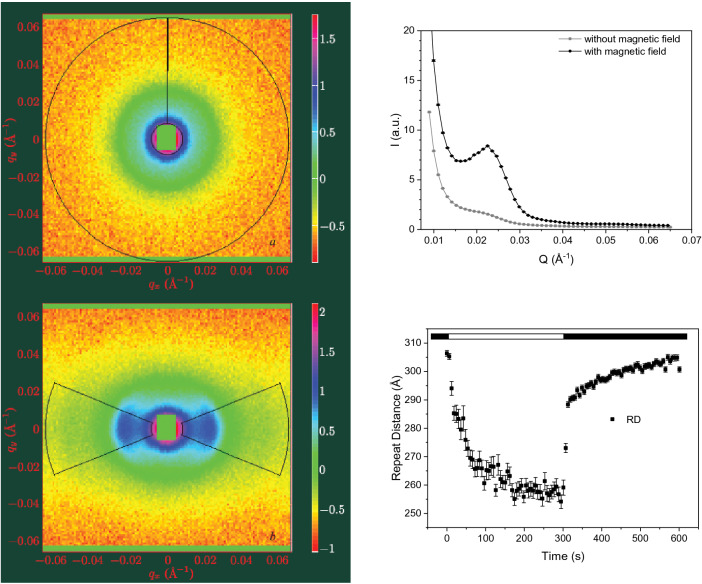
Effect magnetic field on the scattering signal from isolated thylakoid membranes, and light-induced changes in the RD, measured on magnetically aligned membranes. Left: 2D scattering profiles of isolated spinach thylakoid membranes in the absence and presence of 1.5 T magnetic field (upper and lower panels, respectively); black sectors represent the area of radial averaging; colour codes are representing the differential scattering cross-section values in a logarithmic scale in arbitrary units; and Top Right: the corresponding radially averaged SANS profiles. Bottom Right: Typical light-induced RD changes—as indicated by the light (light on) and dark (light off) horizontal bars. RD values were calculated from RD = 2*π*/Q*, where the Q* peak position was obtained by fitting the 1D curves with the sum of a power function and a Gaussian (Nagy et al. 2013). Illustration composed from figures published in (Nagy et al. 2011) and (Nagy et al. 2014a). Measurements were performed on the D22 SANS instrument at the Institut Laue-Langevin (sample-to-detector distance = 8 m, collimation = 8 m, *λ* = 6 Å)

Of particular interest, the magnetic orientation of thylakoid membranes which can be achieved on isolated plant thylakoids (Sadler and Worcester 1982) and also on some algal cells with inherently anisotropic thylakoid arrangements (Nagy et al. 2012) significantly enhances the scattering intensity. Thylakoid membranes, due to their diamagnetic anisotropy, are aligned in an external magnetic field of ~ 0.5–1.5 T with their membrane normals tending to be parallel to the direction of the magnetic field (Knox and Davidovich 1978; Garab 1996). The edge-alignment of thylakoid membranes favours the Bragg diffraction and narrows the azimuthal angle of the ’useful’ signal (Fig. 1a) which improve the S/N ratio. It has also been shown that the magnetic field exerts no effect on the peak position (Fig. 1b), ruling out magnetic-field-induced artefact in RD. The improved S/N ratio allowed observing rapid membrane reorganizations with time resolution of several seconds (Fig. 1c) (Nagy et al. 2011). Without magnetic alignment of the membranes, the time resolution of the experiments is evidently lower. Nonetheless, time-resolved experiments are also feasible on intact systems such as live algal cells (Nagy et al. 2011, 2014b), cyanobacteria (Nagy et al. 2011; Liberton et al. 2013a, b) or entire leaf segments (Ünnep et al. 2014b).

**Fig. 2:**
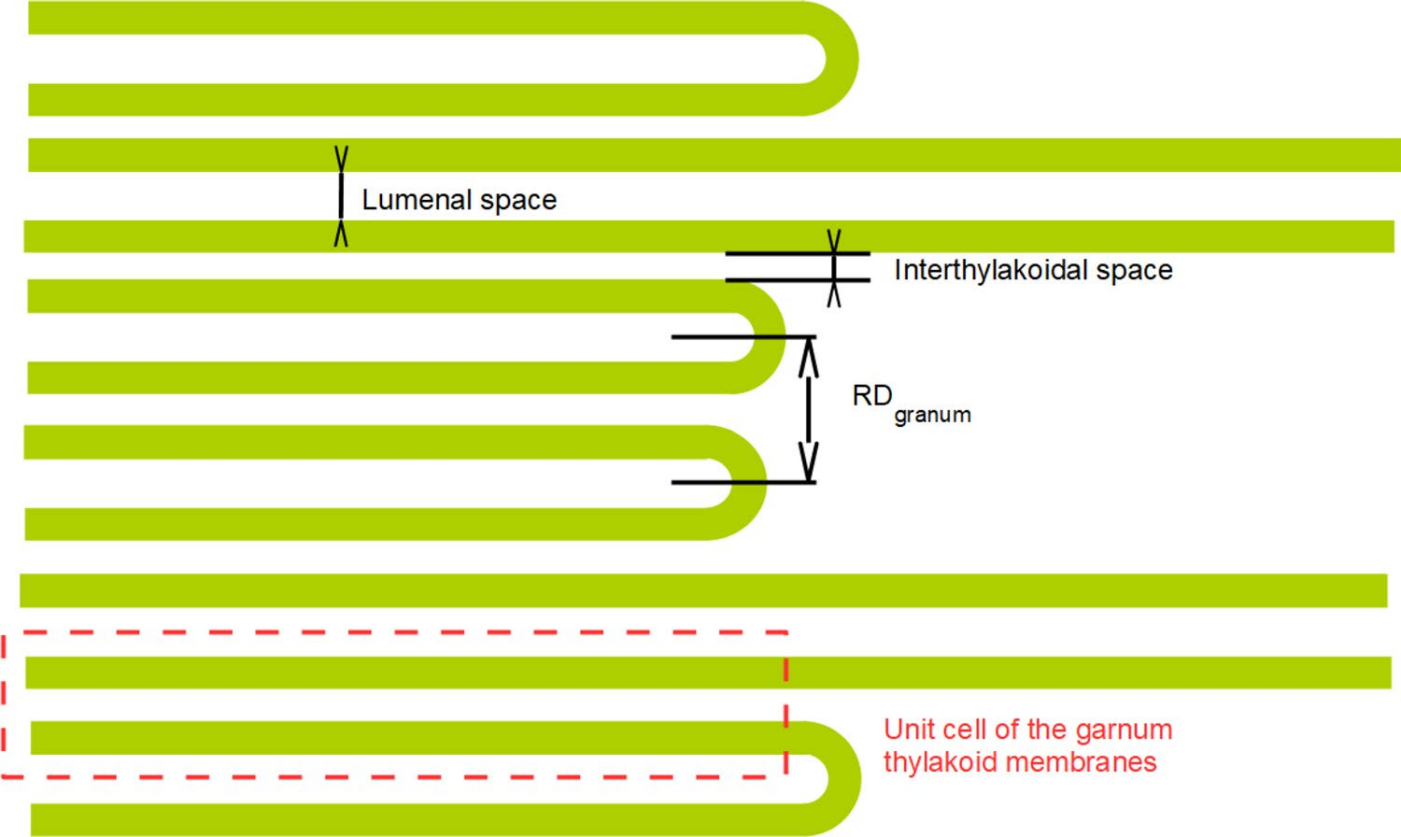
Schematic representation of the granum-stroma thylakoid membrane assembly of higher plants, showing the main structural parameters. For clarity, the arrangement of the main protein complexes in the thylakoid membranes is not displayed in the figure (see, e.g. (Dekker and Boekema 2005) or (Nagy et al. 2014b)). The form factor of grana is determined by the scattering length density distribution of the two bilayer membranes and the embedded protein compounds (mainly LHCII and PSII), together with their protrusions in the luminal and the interthylakoidal aqueous phases

At the present state of the research field, the primary information derived from the SANS curves of thylakoid membranes is mostly confined to the repeat distance (RD) of thylakoids (cf. Fig. 2). This information can be deduced from the first-order Bragg peak of the scattering curves. The RD values, calculated from the Q* peak positions of the scattering vector of the SANS profile and the RDs, obtained from electron microscopy (EM), are in reasonable agreement with each other (Ünnep et al. 2014b). Also, variations in the peak positions, shifts due to shrinking or swelling of the membranes are in harmony with complementary data or are perfectly in line with the expectations (e.g. upon variations in the osmotic strength in algal cells (Nagy et al. 2012) or isolated plant thylakoid membranes (Posselt et al. 2012)). Diminishment or the absence of this peak has also been correlated with the decreased lamellar order. Further, by eliminating the periodic structures via using isolated thylakoid membranes suspended in hypotonic low-salt media, led to the disappearance of the Bragg peak, while retaining the functional thylakoid membrane (Holm 2004). (Please note that the original assignment of the Bragg peak as stroma thylakoids was in error—which was corrected upon more systematic SANS and EM investigations on isolated thylakoid membranes and plant leaves (Ünnep et al. 2014b)). Also, changes in the shape of the scattering profiles (e.g. broadening or sharpening of the bands due to e.g. variations in the mosaicity of the membranes) are in harmony with other data characterizing the sample (Ünnep et al. 2017 and references therein).

The experiments referenced above, in general, justify the use of the simple approach using the first-order Bragg peak and drawing conclusions on the absence or presence of periodic order and on the RD values. However, there are possible complications due to the fact that, in general, no information is available on the form factor, *F*(*Q*). The form factor is the Fourier transform of the scattering length density distribution of the unit cell (here, the thylakoid). The lattice formation of thylakoid membranes is described by the so-called structure factor, *S*(*Q*), and the scattering intensity is given by the product of |*F*(*Q*)|^2^*S*(*Q*) (see e.g. (Ünnep et al. 2014a). Hence, strictly speaking, the variations of both the form factor and the structure factor must be taken into consideration. Trivial cases such as the absence of periodicity or disruption of membranes and changes dominated by shrinking or swelling can be treated easily; in all other cases, complementary techniques and reasonable assumptions (such as negligible changes in the form factor) can help the interpretation of data. In many cases, we have no reason to presume significant variations in the form factor, and thus conclusions can be drawn on perturbations of the periodic order of the thylakoids and/or on changes in their RDs (Nagy et al. 2014b; Karlsson et al. 2015; Herdean et al. 2016).

The first-order Bragg peak is generally observable around 0.02–0.03 Å^−1^ in isolated plant thylakoid membranes and leaves (Ünnep et al. 2014b), 0.03–0.04 Å^−1^ in diatom (Nagy et al. 2012) and green algae (Nagy et al. 2014b), and in the 0.01–0.035 Å^−1^ range (largely depending on the phycobilisome antenna mutation of the individual organism) for cyanobacteria (Nagy et al. 2011; Liberton et al. 2013b; Ünnep et al. 2014a; Jakubauskas et al. 2019). It is well known that there are additional peaks at higher Q values in all thylakoid-containing samples, cyanobacteria, algal cells and higher plants as well as in isolated plant thylakoid membranes. In some cases, the additional peaks could be assigned to 2nd and higher-order Bragg peaks (Ünnep et al. 2014b; Bar Eyal et al. 2017). However, in most publications, the authors focus on the first-order Bragg peak, and avoid discussing the origin of the additional signals—evidently because of the high complexity of the membranes and of possible other sources of scattering from ordered molecular arrays, e.g. cell walls, starch and other cell organelles. In general, efforts must be made to rule out contributions from such structures other than thylakoid membranes; this has been done by using leaf segments containing no mature chloroplasts (Ünnep et al. 2014b). Also, starch can be degraded by dark adaptation of leaves. In some works, association of different diffraction peaks with RDs of different sub-populations of the cyanobacterial thylakoid membranes was also proposed (Liberton et al. 2013b)—though this is not supported by a more recent analysis (Jakubauskas et al. 2019).

A comprehensive interpretation of data has recently been presented, which is based on an estimated scattering length density distribution of a unit cell of thylakoid membranes (allowing the determination of the form factor). In this model, the thylakoids are represented as a bilayer containing intrinsic proteins, and inner (luminal) and outer aqueous phases, also containing proteins and protruding polypeptide sections. The presented advanced mathematical model, which uses different stacked layers with variable layer number and membrane rigidity (as the structure factor), demonstrated that the periodic order of the thylakoid membranes in different cyanobacterial cells could be simulated with reasonable precision (Jakubauskas et al. 2019). Deeper understanding of the scattering signal from the multilamellar cyanobacterial thylakoid membrane system could be further improved if the basic structural unit of the system could be independently studied. This would allow to measure directly the unit cell form factor of the thylakoids. This kind of cyanobacterial preparation, to our knowledge is presently not available, and certainly has not been studied by SANS. Also, some of the derived information (e.g. the large variation in membrane thickness) will require further verification, and independent experimental confirmation. Nevertheless, the approach outlined by Jakubauskas and coworkers will help the research community to expand the information deduced from SANS experiments on photosynthetic membranes. Application of the presented model for higher organisms and adopting this approach for treating scattering data upon physicochemical stress responses will most certainly lead to a fruitful synthesis of different approaches in the field.

The full mathematical model of the scattering profile from a stacked double bilayer membrane system of thylakoids (Jakubauskas et al. 2019), applied for cyanobacteria, can, in principle, be extended to algal cells and leaves and isolated thylakoids of vascular plants. While these membrane systems are more complex, they display much clearer scattering profiles, than most cyanobacteria; with much better defined diffraction peaks and with less problem of reproducibility that was experienced by Jakubauskas et al. (2019). Also the unit cell (cf. Fig. 2) form factor of the isolated granum thylakoid membranes can be and has been studied. Indeed, BBY (Berthold et al. 1981) PSII membrane fragments give rise to a scattering signal that almost perfectly reproduces the second characteristic peak of isolated thylakoid membranes, a peak generally observed between 0.05 and 0.07 Å^−1^ in isolated plant thylakoid membranes and leaves (Nagy 2011; Ünnep et al. 2014b). This has been successfully modelled with a stacked pair of membranes (Nagy 2011). Employing the model of Jakubauskas et al. (2019) and extending it to the entire scattering profile would be of great importance. In such a systematic approach, however, the lateral heterogeneity of plant thylakoid membranes complicates the picture: the protein composition of granum and stroma thylakoid membranes as well as their luminal contents differ significantly from each other—thus, two form factors should be defined. In addition, the interthylakoidal (stacking) distances are also substantially larger for the stroma thylakoids than for the grana. With rare exceptions, however, Bragg peaks around 0.015–0.017 Å^−1^ (corresponding to stroma thylakoids with an RD in the range of ~ 420–360 Å) cannot be observed—probably due to the relatively poor periodicity of these thylakoids.

As emphasized by Jakubauskas and coworkers in accordance with similar considerations published earlier, instrument resolution may also exert some influence on the shape (Nagy et al. 2013) and position (Jakubauskas et al. 2019) of the peak. Thus, the interpretation of the experimental results is always to be performed under well-controlled conditions of the measuring device. In most experiments, in which scattering profiles are compared between wild-type and mutant samples and/or changes in the scattering are induced by external factors, such as light, temperature, ionic and osmotic strengths, the effects of instrumental factors can usually be ignored.

Neutron scattering (diffraction) studies on reconstituted thylakoid lipid systems, also advanced our understanding on the self-assembly of thylakoid membranes. Experiments using different combinations of the four lipid constituents of thylakoid membranes deposited on silicon wafers and measured at varying relative humidity conditions highlighted the importance of bilayer and non-bilayer lipids and lipid phases (Demé et al. 2014). These experiments demonstrated that both the relative amounts of the non-bilayer lipid species and the relative humidity of the sample determined the formation and stabilization of the lamellar structure.

## Structural dynamics of photosynthetic samples

Different neutron scattering spectroscopy techniques can be used to probe the dynamics of different samples on a broad range of time- and lengthscales from the fs to ~ 100 ns and from below Å to dozens of nanometers, respectively (Sokolov and Sakai 2012). The majority of earlier structural dynamics studies were based on elastic incoherent (EINS) or quasielastic neutron scattering (QENS) measurements (e.g. (Pieper et al. 2007; Pieper and Renger 2009)) or on inelastic neutron scattering (e.g. (Pieper et al. 2004, 2012)). These studies were reviewed in Nagy et al. (2014a). Recently, the studies presented in Pieper et al. (2004) were extended to characterize LHC II vibrations at physiological temperatures, which contributed to the better understanding of ultrafast excitation energy transfer processes in this antenna complex (Golub et al. 2015). Further QENS studies of native and mutant (lacking the Chl *a* 612 pigment molecule) LHCII, coupled with absorption and fluorescence measurements, indicated correlation between the presence of conformational dynamics and the position of the excited electronic states in the antenna complex (Vrandecic et al. 2015). The effect of the oligomeric state of the LHCII on the dynamics of this protein was also investigated up to physiologically relevant temperatures (Golub et al. 2018).

In the past few years, neutron spectroscopy techniques were employed to study the internal dynamics of intact photosynthetic organisms. Russo and coworkers with the help of EINS and QENS were able to demonstrate that in the green alga *Chlamydomonas reinhardtii* mutations in the plastoquinone—(PQ)-binding niche of the D1 protein of Photosystem II (PSII)—resulting in impaired electron transport—influence the dynamics of the thylakoid membranes. Specific amino acids in the PQ-binding niche appeared to be essential for preserving a more rigid environment, required for efficient electron transport from Q_A_ to Q_B_ (Russo et al. 2019).

Neutron spin echo (NSE) experiments also provided a unique insight into the dynamics of thylakoid membranes in live cyanobacteria. Stingaciu and coworkers carried out the first in vivo measurements using NSE on *Synechocystis* sp. PCC 6803 cells, and observed significant changes in the structural-dynamics parameters, attributed to undulation or shape fluctuations of the thylakoid membranes (Stingaciu et al. 2016). In particular, illumination appeared to rigidify the thylakoid membranes compared to those in dark-adapted cells, a clear connection between membrane mobility and photosynthetic activity. It was also shown that the membranes after illumination largely retained their flexibility in the presence of PSII inhibitor DCMU, pointing to the role of transmembrane electrochemical potential gradient for protons (Δμ_H+_) in determining the mechanical properties of thylakoid membranes. (DCMU, (3-(3,4-dichlorophenyl)-1,1-dimethylurea).)

NSE measurements also revealed that binding of DCMU per se in dark-adapted cells also affected significantly the membrane flexibility (Stingaciu et al. 2019). This unexpected observation is difficult to explain in terms of the known inhibitory effects of DCMU. However, similar anomaly has been observed in the diatom alga *Phaeodactylum tricornutum*, in which DCMU did not block the light-induced swelling of thylakoid membranes but prevented the recovery of the membrane reorganizations upon dark-readaptation (Nagy 2011). Clarification of these observations requires further studies. Replacing the PQ by DCMU at the *Q*_B_ site might exert an effect on the structurally flexible region of the protein moiety of PSII; this step might change the electrostatics of the membranes on the stromal side, thus affecting the stacking interactions.

In general, NSE data demonstrated the “active function [of thylakoid membranes] during energy conversion, rather than a rigid support frame for other photosynthetic components” (Stingaciu et al. 2019). Ünnep et al., based on SANS measurements, also concluded that the thylakoid membrane systems appear to “actively participate in the energy conversion steps and in different regulatory functions” (Ünnep et al. 2017). NS techniques are most suitable to reveal details of this active role of thylakoids, which is evidently based on their remarkable inherent plasticity.

## Neutron protein crystallography

Atomic resolution structural information—most commonly obtained via X-ray crystallography—is indispensable for understanding the function of biological macromolecules and protein complexes. However, precise position of hydrogen atoms—an often critical information e.g. understanding enzyme operations—is rarely provided by this technique. In contrast, neutron crystallography can locate key hydrogen atoms (or protons) in the protein structure previously determined by complementary techniques. Unfortunately, to this date, the number of protein structures determined via neutron crystallography is very limited, mostly due to flux limitations at existing neutron sources and due to the small number of neutron crystallography beamlines and the limited beam-time availability. Collecting neutron crystallography data from membrane proteins is especially challenging, due to the large crystal size required for the experiments—challenging to be grown from membrane proteins, which poses extra limitation on the study of proteins relevant in the light reactions of photosynthesis—almost exclusively taking place in the thylakoid membranes.

**Fig. 3:**
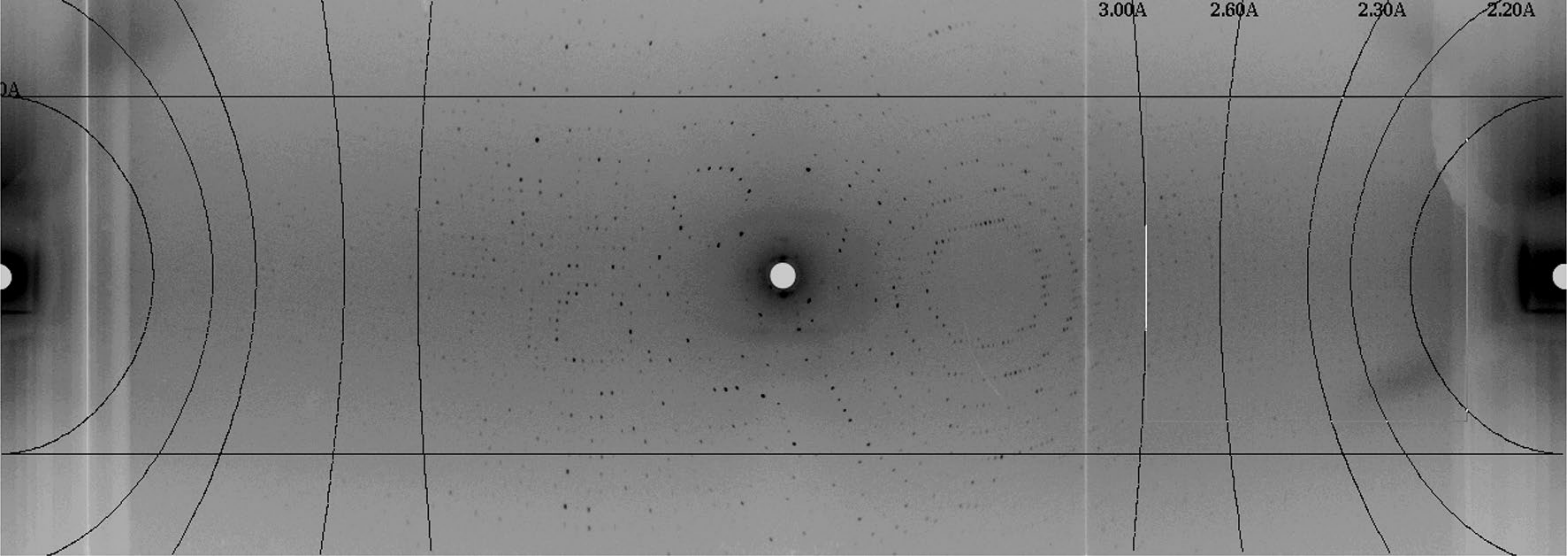
Diffraction pattern from the RuBisCO enzyme, collected on the BIODIFF beamline at the Forschungs-Neutronenquelle Heinz Maier-Leibnitz (FRMII/MLZ) in Garching, Germany. Personal communication, courtesy of Dr. Marvin Seibert (Uppsala University) and Dr. Andreas Ostermann (MLZ)

Nevertheless, recent advancements in neutron macromolecular instrumentation—see e.g. (Meilleur et al. 2018)—and in available crystal size and quality pave the way for the first results. Neutron analysis of crystals of the Fenna–Matthews–Olson complex from a green sulphur bacteria will help to understand how different bacteriochlorophylls in the complex are imparted with their own particular site energy by their chemical environment (Lu et al. 2019). Neutron diffraction experiments on more complex systems such as PSII crystals are also demonstrating the advancement of the field (Hussein et al. 2018). Marvin Seibert (Uppsala University, Sweden) and collaborators are presently employing neutron crystallography to better understand the catalytic process of carbon fixation, via performing neutron crystallography experiments to determine the protein structure of a RuBisCO sample obtained from spinach leaves, specifically H-positions in the enzyme’s active site (Fig. 3).

Apparently, the state-of-the-art technology is at the brink of addressing substantial questions in photosynthesis research, thus the next-generation instruments proposed (Coates and Robertson 2017) or under construction (see NMX—Macromolecular diffractometer in (Andersen et al. 2020; Markó et al. 2020)) will allow to perform experiments on significantly smaller crystals, and are expected to answer a wide range of scientific questions in the field.

## Concluding remarks

In this mini-review, our major aim was to show how neutron scattering techniques have advanced our knowledge about the structure, flexibility and dynamics of thylakoid membranes and other photosynthetic preparations. These techniques provide unique information on in vitro and in vivo systems and—when combined with complementary techniques of structural biology and functional measurements—will most certainly help testing the physiological performance of crop plants, and by this means, help designing and constructing or breeding plants with improved stress tolerance and/or productivity.

## Abbreviations

BBY: Photosystem II membrane fragments
CD: Circular dichroism
Chl: Chlorophyll
DCMU: (3-(3,4-Dichlorophenyl)-1,1-dimethylurea)
EINS: Elastic incoherent neutron scattering
HWHM: Half-width at half maximum
INS: Inelastic neutron scattering
LHC II: Light-harvesting complex II
NS: Neutron scattering
NSE: Neutron spin echo
PBS: Phycobilisome
psi: Polymer and salt induced
PS I: Photosystem I
PS II: Photosystem II
RD: Repeat distance
QENS: Quasielastic neutron scattering
Q: Scattering vector
SANS: Small-angle neutron scattering
